# Development, validation and clinical application of a method for the simultaneous quantification of lamivudine, emtricitabine and tenofovir in dried blood and dried breast milk spots using LC–MS/MS

**DOI:** 10.1016/j.jchromb.2017.06.012

**Published:** 2017-08-15

**Authors:** Catriona Waitt, Sujan Diliiy Penchala, Adeniyi Olagunju, Alieu Amara, Laura Else, Mohammed Lamorde, Saye Khoo

**Affiliations:** a Department of HIV Pharmacology, University of Liverpool, 70 Pembroke Place, Liverpool, L69 3GF, UK; b Infectious Diseases Institute, Makerere University College of Health Sciences, Kampala, Uganda; c Royal Liverpool University Hospital, Prescot Street, Liverpool, L7 8XP, UK; d Faculty of Pharmacy, Obafemi Awolowo University, Ife-Ife, Nigeria

**Keywords:** Liquid chromatography, Mass spectrometry, Antiretroviral

## Abstract

**Objectives:**

To present the validation and clinical application of a LC–MS/MS method for the quantification of lamivudine (3TC), emtricitabine (FTC) and tenofovir (TFV) in dried blood spots (DBS) and dried breast milk spots (DBMS).

**Methods:**

DBS and DBMS were prepared from 50 and 30 μL of drug-spiked whole blood and human breast milk, respectively. Following extraction with acetonitrile and water, chromatographic separation utilised a Synergi polar column with a gradient mobile phase program consisting of 0.1% formic acid in water and 0.1% formic acid in acetonitrile. Detection and quantification was performed using a TSQ Quantum Ultra triple quadrupole mass spectrometer. The analytical method was used to evaluate NRTI drug levels in HIV-positive nursing mothers-infant pairs.

**Results:**

The assay was validated over the concentration range of 16.6–5000 ng/mL for 3TC, FTC and TFV in DBS and DBMS except for TFV in DBMS where linearity was established from 4.2–1250 ng/mL. Intra and inter-day precision (%CV) ranged from 3.5–8.7 and accuracy was within 15% for all analytes in both matrices. The mean recovery in DBS was >61% and in DBMS >43% for all three analytes. Matrix effect was insignificant.

Median AUC_0-8_ values in maternal DBS and DBMS, respectively, were 4683 (4165–6057) and 6050 (5217–6417) ng h/mL for 3TC, 3312 (2259–4312) and 4853 (4124–6691) ng h/mL for FTC and 1559 (930–1915) and 56 (45–80) ng h/mL for TFV. 3TC and FTC were quantifiable (>16.6 ng/mL) in DBS from 2/6 and 1/6 infants respectively whereas TFV was undetectable in all infants.

**Conclusions:**

DBS and DBMS sampling for bioanalysis of 3TC, FTC and TFV is straightforward, robust, accurate and precise, and ideal for use in low-resource settings.

## Highlights

• We report an LC–MS/MS method for quantitation of 3TC, FTC and TFV in blood and breast milk.
• Agreement between dried blood and plasma measurement of 3TC and TFV is good.
• 3TC and FTC reach high concentrations in breast milk.
• 3TC and FTC are measurable in a significant proportion of breastfed infants.

## 1 Introduction

It is internationally recommended that HIV-positive women receive triple antiretroviral therapy (ART) throughout pregnancy until the end of breastfeeding or for life irrespective of clinical disease stage or CD4 count [1]. As breastfeeding remains the only acceptable, feasible, affordable, sustainable and safe infant feeding option in many parts of the world [2], the number of infants exposed to antiretroviral drugs through pregnancy and breastfeeding will continue to increase.

First-line ART comprises efavirenz (EFV), tenofovir diproxil fumarate (TDF) and either lamivudine (3TC) or emtricitabine (FTC) used preferably as a fixed-dose combination. It is important to understand the breast milk transfer of these drugs since low infant levels predispose to HIV-drug resistance should HIV transmission occur [3], [4], and there is conflicting data regarding the effects of tenofovir (TFV) on developing bone [5]. The pharmacokinetic profiles in paired maternal and infant plasma (calculated from dried blood spots [DBS]) and breast milk (BM) have been reported for EFV [6] and 3TC [7], but only three studies have sought to measure TFV [8], [9], [10] and a single study FTC in the BM of HIV-positive mothers [8], and these did not present intensive pharmacokinetic profiles and paired mother and infant data.

Furthermore, a systematic review of antiretroviral measurement in BM noted marked methodological differences with regard to the collection and storage of samples, the matrix used to prepare the standards and quality controls, the fraction of milk analysed, the extraction method and the type of internal standard used [11]. We have recently developed and validated dried blood spot (DBS) and dried breast milk spot (DBMS) methodology for EFV [12] and nevirapine (NVP) [13]. In addition to quantifying drugs in whole BM, these techniques have the advantage of being suitable for collection and storage in low resource settings. We now report the DBS and DBMS LC–MS/MS method for accurate simultaneous quantitation of TFV, 3TC and FTC, with application of the method in breastfeeding mother-infant pairs.

## 2 Materials and methods

Lamivudine (3TC) and internal standard (IS) lamivudine-^15^N_2_,^13^C (3TC-IS), emtricitabine (FTC) and emtricitabine-^13^C,^15^N_2_ (FTC-IS) and tenofovir (TFV) were obtained from TRC Canada (North York, Ontario). LC–MS grade acetonitrile was obtained from Fisher Scientific (Loughborough, Leicestershire, UK), methanol from VWR International (Lutterworth, Leicestershire, UK), formic acid from Sigma-Aldrich (Gillingham, Dorset, UK) and water was produced from an Elga Option 4 water purifier (Elga LabWater, High Wycombe, Buckinghamshire, UK) and was further purified to 18.2 MΩ with a Purelab Classic UVF (Elga LabWater, High Wycombe, Buckinghamshire, UK). Whatman 903 Protein Saver cards were obtained from Scientific Laboratory Supplies (Hessle, East Yorkshire, UK). Blank whole blood was collected into EDTA tubes from drug-free healthy volunteers and blank BM samples were obtained from the Wirral Mothers’ Milk Bank, Clatterbridge Hospital, Wirral, UK; the University of Liverpool Research Ethics Committee approved these processes.

### 2.1 LC–MS systems and conditions

The LC–MS system consisted of a Synergi polar-RP column (80A, 150 *2.0 mm and 4 μ; Phenomenex, Macclesfield, UK) with a 2 μm C_18_ Quest column-saver (Thermo Electron Corporation, Hemel Hempstead, Hertfordshire, UK) on a HPLC connected to a TSQ Quantum Ultra triple quadrupole mass spectrometer (Thermo Electron Corporation, Hemel Hempstead, Hertfordshire, UK) equipped with a heated electrospray ionisation source (H-ESI). Xcalibur Software and LCquan (version 2.6.1, Thermo Fisher Scientific, Hemel Hempstead, UK) were used for method setup, data acquisition, data processing and reporting.

A solvent gradient programme (flow rate of 400 μL/min) with 0.1% formic acid in water (mobile phase A) and 0.1% formic acid in acetonitrile (mobile phase B) was used for chromatographic separation. The gradient programme started with 95% mobile phase A, and held for 0.2 min. Mobile phase A was decreased to 70% over 0.5 min. This was maintained for 3.0 min, followed by column equilibration to the initial conditions. The total run time was 6.0 min. The Injection volume was 25 μL and the needle was washed twice with 2 mL water: acetonitrile (95:5) between injections. The MS was operated in positive ionisation mode to produce characteristic fragmentation patterns. The electrospray voltage was set at 4.0 kV, the capillary temperature at 300 °C and the vaporizer temperature at 350 °C. The sheath and auxiliary gas pressures were set of 50 and 10 arbitrary units, respectively. Argon was used as the collision gas at a pressure of 1.5 mTorr. Product ion characterisation and tuning was done by directly infusing 1 μg/mL solutions of all the three analytes and their internal standards separately into the MS using a syringe at a flow rate of 5 μL/min. The most sensitive mass transitions (*m*/*z*) were monitored in selective reaction monitoring.

### 2.2 Stock solutions, calibration standards and quality controls (QC)

3TC and FTC stock solutions were prepared from their respective reference standards in 100% methanol, and TFV in 100% water to obtain a final concentration of 1 mg/mL and refrigerated at 4 °C until use. Similarly, 3TC-IS and FTC-IS 1 mg/mL stock solutions were prepared and frozen at −40 °C; working stock solutions of 250 ng/mL were prepared in methanol-water (50:50 v/v) and refrigerated at 4 °C prior to use. At the time of validation, the cost to purchase a stable isotope labelled internal standard for TFV was beyond our budget. 3TC-IS, FTC-IS and an analog IS (2-Chloroadenosine) were evaluated as potential IS for TFV in pre-validation experiments. FTC-IS was selected on the basis of it exhibiting a consistent response. A whole blood working stock containing all three analytes at 10 μg/mL was prepared, tumbled for 60 min and used to make nine whole blood standards in the range of 16.6–5000 ng/mL by serial dilution, including a blank sample of blood alone. Whole blood low quality control (LQC, 40 ng/mL), medium quality control (MQC, 400 ng/mL) and high quality control (HQC, 4000 ng/mL) samples were similarly prepared from a 10 μg/mL working stock. Working stocks in human breast milk were similarly prepared, with the difference that the calibration range for tenofovir was 4.2–1250 ng/mL with LQC 10 ng/mL, MQC 40 ng/mL and HQC 400 ng/mL respectively due to the lower anticipated concentrations of this analyte in clinical specimens [9].

### 2.3 DBS and DBMS standard and QC preparation

DBS standards and QCs were prepared by carefully spotting exactly 50 μL of whole blood standards and QCs on each circle of Whatman 903 Protein Saver Cards. DBMS standards and QCs were similarly prepared by spotting 30 μL of breast milk standards and QCs. Spotted cards were left to dry at room temperature overnight and stored with dessicant sachets in ziplock bags at −80 °C.

### 2.4 Sample pre-treatment

The entire DBS or DBMS spot was removed using a 12 mm hole punch and folded into a 7 mL screw cap tube. For DBS, initial extraction was with 200 μL 0.1% formic acid in water for 5 min prior to the addition of 3TC-IS and FTC-IS. IS was added to the extraction solvent [14] since spotting directly onto the card was not feasible for samples collected under field conditions. Then 800 μL acetonitrile was added to each tube and after vortexing, tubes were centrifuged at 4000 rpm for 10 min. 850 μL of each sample was carefully pipetted into a 5 mL tube, before evaporation to dryness under a stream of nitrogen. Finally, samples were reconstituted in 100 μL water: acetonitrile (99:1 v/v) and transferred into autosampler vials.

DBMS samples were extracted with 1 mL of acetonitrile: water (70:30, v/v) by tumbling for 30 min in the presence of 3TC-IS and FTC-IS. 800 ul was then transferred to a 5 mL glass tube, before evaporation to dryness under a stream of nitrogen. Finally, samples were reconstituted in 100 μL water: acetonitrile (99:1 v/v) and transferred into autosampler vials.

### 2.5 Calibration curves, accuracy and precision

A calibration curve consisting of a zero blank, nine standards in the range of 16.6–5000 ng/mL (and 4.2–1250 ng/mL in the case of DBMS for TFV) (n = 2 separate extractions for each level) and QCs (n = 6 separate extractions for each level) were run for each of DBS and DBMS. Calibration curves were constructed using a linear regression equation of analyte/IS peak area ratios versus nominal concentrations with a 1/concentration weighting. Accuracy was defined as percentage deviation of measured concentration from the nominal value and precision was defined as the percentage coefficient of variation (%CV). Not less than 75% of all standards and 67% of all QCs (50% at each level) in any batch were required to have a percentage deviation within ±15%.

### 2.6 Recovery, matrix effect and dilution integrity

The percentage recovery and matrix effect were determined quantitatively (in accordance to the recommendations of Matuszewski et al) by preparing LQC, MQC and HQC samples (n = 6), DBS and DBMS, as follows: A: pure standard solutions of analytes in mobile phase, directly injected onto the column; B: blank DBS/DBMS extracts, spiked with analytes after extraction; C: whole blood and BM samples spiked with analyte before extraction [15].

The overall recovery (process efficiency; PE) was defined as the ratio of the absolute peak-area response of the analytes in whole blood/BM spiked with drug prior to extraction (C) to the peak area response of analytes in an aqueous mobile phase sample (A) (C/A × 100). The matrix effect (ME) was calculated as the ratio of the peak area response of analytes spiked into blank DBS/DBMS extracts after extraction (B) to the peak areas of the analytes in mobile phase (A) (B/A × 100). The recovery (extraction yield, RE) was derived from the absolute peak-area response in whole blood and BM samples spiked with analytes prior to extraction (C), expressed as the percentage of the response of the equivalent amount of analyte spiked into DBS/DBMS extracts after extraction (B) (C/B × 100). A %CV of ≤15% across all QC concentrations was set as the level of acceptance for both recovery and matrix effect in line with the FDA method validation guidelines [16].

To investigate dilution integrity for clinical samples with concentrations above the reference range, 50 μL of whole blood containing 9000 ng/mL of 3TC, FTC and TFV and 30 μL of breast milk containing 9000 ng/mL of 3TC and FTC and 2000 ng/mL of TFV were spotted onto each circle of Whatman 903 cards. The samples were dried and extracted as described previously. The extracts were diluted 2× and 4× using blank DBS and DBMS spots similarly extracted in the same batch. The final concentrations were then derived by back-calculating with the appropriate dilution factor.

### 2.7 Stability and re-injection (inter-day and intra-day) reproducibility

We evaluated the short-term and medium-term stability of 3TC, FTC and TFV in DBS and DBMS at different storage and processing conditions. This was evaluated by storing extracted QC samples at room temperature and in the autosampler (4 °C) for 24 h. Both inter-day and intra-day reproducibility were evaluated. For medium and long-term stability at room temperature (24 °C), DBS and DBMS QC samples were stored at room temperature for 1 and 6 months (DBS) and 9 months (DBMS). Our research laboratories in the UK, Nigeria and Uganda were all temperature controlled, without significant fluctuations in ambient temperature. Drug concentrations in stored samples were read off a calibration curve using freshly made standards and QCs. An accepted validation assay batch (standards and QCs) was re-injected after 24 h in the autosampler to evaluate re-injection reproducibility. Samples were considered stable if values were within the acceptance limits of accuracy (±15% of their respective nominal concentration) and precision (≤15%CV).

### 2.8 Effect of haematocrit

60 mL whole blood was collected in EDTA and prepared following the method of Koster et al. [17]. Centrifugation at 15 g for 15 min at room temperature separated the plasma and red blood cells. Increasing volumes of plasma were added to whole blood to ‘dilute’ it, providing decreasing HCT levels of approximately 100%, 80%, 60%, 40%, 20% and 0% of ‘normal’, respectively. The non-centrifuged whole blood served as 100% control, the plasma as 0% and a >100% HCT sample was produced by adding 500 μL of packed cells to 1 mL of whole blood. These were then analysed for%HCT levels on a UniCel DxH 800 Workcell auto-analyser (Beckman Coulter Ltd, High Wycombe, United Kingdom). The blood was then spiked with the three analytes to provide the LQC, MQC and HQC levels as detailed above (including tumbling for 60 min) prior to spotting 50 μL of each onto Whatman 903 cards. These samples were then dried, extracted and analysed. Furthermore, concentrations of 3TC and TFV were measured in paired plasma and DBS from 6 nursing mothers in Uganda (Section 2.9), and the relationship between the maternal plasma: DBS ratio and HCT considered.

### 2.9 Clinical application

The validated method was applied in two studies to evaluate DBS and BM pharmacokinetic profiles of ARVs in HIV-positive nursing mothers and their infants.

3TC was measured in Ugandan HIV-positive nursing mothers (n = 6) receiving regimens containing 150 mg taken every 12 h. Trough concentrations of TFV were measured in a further 6 nursing mothers taking EFV-based ART each evening. Patients were recruited at the Infectious Diseases Institute, Makerere University, Kampala, Uganda. The study protocol and the material transfer agreement were approved by the University of Liverpool Research Ethics Committee, UK and the Joint Clinical Research Centre IRB, Uganda and the Uganda National Council for Science and Technology (HS1675). TFV and FTC were measured in Nigerian HIV-positive nursing mothers (n = 6) recruited from Bishop Murray Medical Centre, Benue State, Nigeria, a cohort which has been reported in detail elsewhere [18]. Ethical approval was obtained from the National Health Research and Ethics Committee (NHREC), Abuja and Obafemi Awolowo University Teaching Hospitals (OAUTHC) Ethics and Research Committee, Ife-Ife, Nigeria.

Paired maternal DBS (mDBS) and DBMS were collected prior to and at 1, 2, 4, 6 and 8 h (3TC, Ugandan cohort), or 0.5, 1, 2, 4, 8 and 12 h (Nigerian cohort) following an observed dose of the drug, taken after a standard breakfast. Ugandan subjects on TFV-containing regimens had trough PK concentrations measured at 12, 16 and 20 h post dosing (not observed) to enable comparison between mDBS and plasma. In Uganda, an intravenous cannula was inserted and 2 mL venous whole blood taken at these time points into EDTA tubes. mDBS were prepared by accurately pipetting 50 μL blood onto the Whatman 903 Protein Saver card; plasma was harvested from the remaining sample and stored at −80 °C until shipment, which enable direct comparison of concentrations obtained from mDBS with plasma. In Nigeria, mDBS samples were collected by direct spotting onto the card, following finger-prick with a sterile lancet. Within 2 min of DBS collection, about 5 mL of BM was manually expressed by the mother and 30 μL aliquots were pipetted onto each circle on the Whatman 903 Protein Saver card. DBS samples were collected from infants (iDBS) at 2 h and 8 h post maternal dosing, after sterile skin cleaning and heel prick using a 2 mm safety lancet (BD, Oxford, Oxfordshire, UK). All samples were stored locally at −80 °C, transported to the Department of Molecular and Clinical Pharmacology, University of Liverpool, UK for analysis on dry ice and then kept at −80 °C. This was because the results from Section 2.7 on long-term stability at room temperature were not yet available.

In both cohorts, all infants were breastfed on demand to reflect real-life situations.

The plasma samples from Ugandan women were processed as follows. To 100 μL of plasma sample 20 μL of internal standard mixture 3TC-IS (2.5 μg/mL) and FTC-IS (2.5 μg/mL) were added. 400 μL of acetonitrile was added to precipitate the proteins followed by centrifugation at 4000 rpm for 10 min 300 μL of the above supernatant was removed and evoparated to dryness under nitrogen steam. The dried residue was dissolved in 100 μL of water: acetonitrile (99:1 v/v) and transferred into auto sampler vials. The samples were analysed on a validated LC–MS/MS method [19].

### 2.10 Data analysis

Non-compartmental analysis of pharmacokinetic data was undertaken using WinNonLin (Phoenix, version 6.1; Pharsight Corp., Mountain View, CA).

To evaluate the agreement between mDBS and plasma concentrations of the drugs, the paired mDBS and plasma samples from Ugandan mothers were correlated using linear regression. This andBland Altman analysis was undertaken using GraphPad Prism (version 5.00 for Windows, GraphPad Software, San Diego California USA, www.graphpad.com). Ratios between concentrations measured in breast milk and maternal dried blood samples were calculated arithmetically and are expressed as DBMS:mDBS ratios.

## 3 Results

### 3.1 LC–MS/MS conditions

**Fig. 1:**
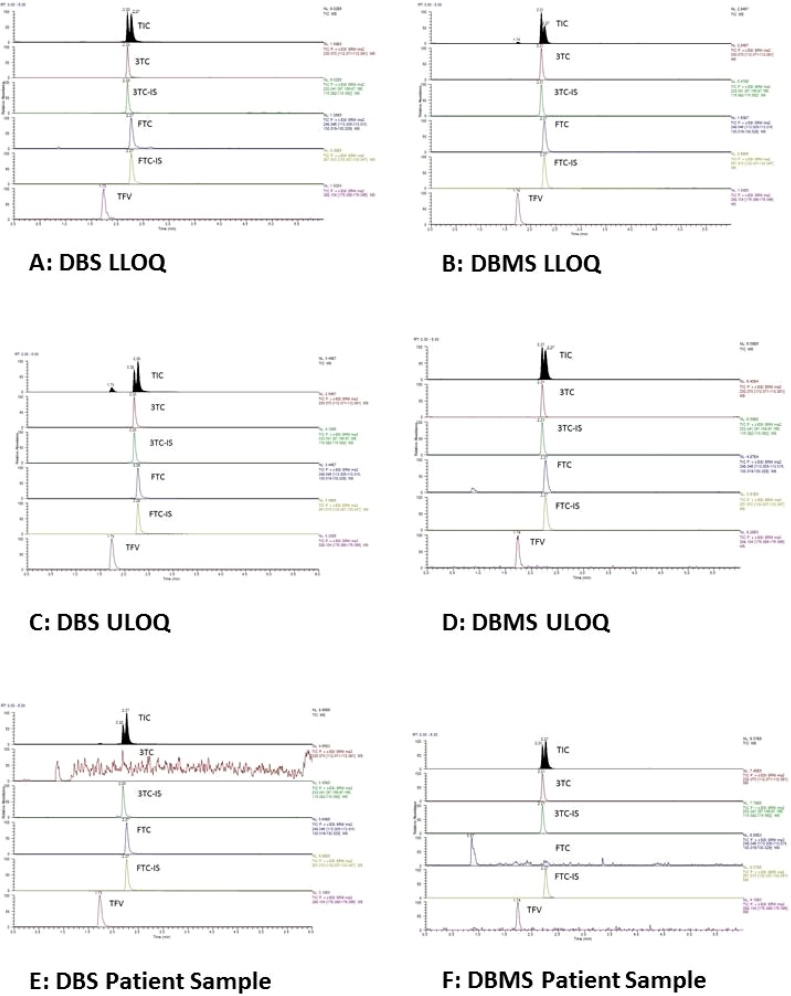
Representative chromatograms at lower and upper limits of quantitation for both DBS and DBMS, together with a representative patient chromatogram.

The transitions (collision energy) were 230.1 → 112.1*m*/*z* (14) for 3TC, 248.0 → 130.1*m*/*z* (15) for FTC, 288.1 → 176.1*m*/*z* (25) for TFV, 233.0 → 115.1*m*/*z* (15) for 3TC-IS and 251.1 → 133.1*m*/*z* for FTC-IS (13). The scan width was set at 0.01 *m*/*z* and the dwell time at 0.01 s. Representative chromatograms are shown in Fig. 1, with a retention times of 2.20 min for 3TC, 2.27 min for FTC, 1.73 for TFV, 2.20 min for 3TC-IS and 2.27 min for FTC-IS. FTC-IS was used as internal standard for quantitation of TFV.

### 3.2 Calibration curves, accuracy and precision (Table 1)

The method was quadratic with weighing factor (1/x^2^) in the range of 16.6–5000 ng/mL for all analytes except TFV DBMS which was linear with weighing factor (1/x) in the range of 4.2–1250 ng/mL, with intra and inter-day accuracy and precision within the acceptance criteria as per FDA and EMA guidelines (Table 1). The mean regression coefficient was >0.99 in both DBS and DBMS.

**Table 1:** Inter-day precision and accuracy for DBS and DBMS assays for TFV, 3TC and FTC.

	Tenofovir (TFV)			Lamivudine (3TC)			Emtricitabine (FTC)		
Nominal value (ng/ml)	mean	Precision (CV%)	accuracy (%)	mean	Precision (CV%)	accuracy (%)	mean	Precision (CV%)	accuracy (%)
DBS (Inter-assay)									
16.6 (LLOQ)	16.6	9.36	−0.09	17.5	6.94	5.48	17.1	5.16	2.69
40 (LQC)	42.2	9.04	5.61	38.1	5.82	−4.83	40.0	5.69	−0.053
400 (MQC)	430.2	8.36	7.56	391.6	4.55	−2.093	394.5	3.68	−1.38
4000 (HQC)	4031.8	7.64	0.79	3753.8	7.76	−6.15	4046.9	5.66	1.17
DBS (Intra-assay)									
16.6 (LLOQ)	16.2	11.2	−2.52	17.3	6.13	4.23	17.0	4.12	2.21
40 (LQC)	41.7	10.9	4.32	38.2	5.77	−4.39	39.7	5.71	−0.88
400 (MQC)	424.9	9.79	6.23	398.1	4.54	−0.49	395.6	3.52	−1.09
4000 (HQC)	4188.5	5.13	4.71	3798.2	8.09	−5.04	4080.9	5.65	2.02

		Tenofovir (TFV)			Lamivudine (3TC)			Emtricitabine (FTC)		
		mean	precision	accuracy (%)	mean	precision	accuracy (%)	mean	precision	accuracy (%)
DMBS (Inter-assay)										
TFV	3TC/FTC									
4.2 (LLOQ)	16.6 (LLOQ)	4.39	16.8	4.41	16.02	11.43	−3.49	16.39	7.22	−1.23
10 (LQC)	40 (LQC)	10.6	18.5	6.43	40.66	17.69	1.64	39.37	12.95	−1.58
40 (MQC)	400 (MQC)	37.23	7.21	−6.93	376.5	4.44	−5.87	372.7	4.66	−6.81
500 (HQC)	4000 (HQC)	512.5	6.43	−6.83	4306.9	4.96	7.67	4257.8	4.10	6.44
DBMS (intra-assay)										
TFV	3TC/FTC									
4.2 (LLOQ)	16.6 (LLOQ)	4.14	12.1	−1.55	16.3	14.8	−1.50	17.5	3.08	5.65
10 (LQC)	40 (LQC)	10.6	9.34	6.13	40.4	7.62	0.95	36.4	8.26	−9.04
40 (MQC)	400 (MQC)	36.7	5.09	−8.28	386.2	2.86	−3.44	376.5	5.01	−5.88
500 (HQC)	4000 (HQC)	503.9	7.67	−8.38	4331.3	6.52	8.28	4228.8	4.86	5.72

### 3.3 Recovery, matrix effect and dilution integrity (Table 2)

The mean (±CV%) percentage (%) recovery (RE), process efficiency (PE) and contribution of the sample matrix (ME) are summarized in Table 2. The mean RE (%CV) of 3TC, FTC and TFV from DBS were 92.2% (8.68), 55.7% (15.0) and 62.8% (9.20), respectively, and from DBMS were 66.1% (14.9), 77.9 (12.6) and 48.6% (14.4), respectively. The percentage ME ranged between 104 and 113% (%CV 2.5-10.2) for the analytes in DBS, and from 83 to 107% (%CV 1–8) for DBMS, respectively.

**Table 2:** Recovery and matrix effect for DBS and DBMS assays for 3TC, FTC and TFV.

Drug	Level	%ME(B/A*100) (SD)	%PE(C/A*100) (SD)	%RE (SD) (C/B*100)
DBS				
TFV	LQC	107.3 (10.21)	61.0 (6.03)	56.9 (2.59)
	MQC	105.1 (3.76)	66.4 (3.02)	63.1 (2.43)
	HQC	101.9 (2.55)	69.8 (1.39)	68.4 (1.71)
	Mean (%CV)	104.8 (2.57)	65.7 (6.70)	62.8 (9.20)
3TC	LQC	106.33 (4.30)	73.09 (7.03)	94.6 (8.11)
	MQC	114.52 (5.35)	90.22 (1.78)	98.7 (2.78)
	HQC	119.96 (6.96)	94.84 (3.79)	83.3 (5.59)
	Mean (%CV)	113.6 (5.54)	86.05 (4.20)	92.2 (8.68)
FTC	LQC	119.4 (8.31)	56.6 (3.30)	46.9 (0.64)
	MQC	113.4 (4.16)	63.3 (2.93)	55.8 (2.49)
	HQC	100.5 (2.82)	64.8(2.17)	64.2 (1.41)
	Mean (%CV)	111.3 (8.45)	61.6 (7.11)	55.7 (15.0)
DBMS				
TFV	LQC	101.3 (4.97)	59.46 (10.0)	53.1 (5.89)
	MQC	113.1 (5.79)	44.89 (3.79)	40.3 (3.30)
	HQC	108.1 (4.97)	56.52 (4.72)	52.3 (3.87)
	Mean (%CV)	107.5 (5.51)	53.6 (14.4)	48.6 (14.7)
3TC	LQC	97.5(7.13)	57.9 (1.48)	59.6 (6.21)
	MQC	98.5 (8.04)	60.3 (4.76)	61.2 (3.64)
	HQC	99.3(6.89)	75.2 (3.55)	77.6 (7.63)
	Mean (%CV)	98.5 (0.93)	64.5 (14.5)	66.1 (14.9)
FTC	LQC	88.1 (4.08)	71.9 (7.58)	80.2 (6.35)
	MQC	84.7 (7.53)	54.5 (7.40)	67.2 (8.09)
	HQC	79.1 (4.94)	68.3 (9.09)	86.4 (11.5)
	Mean (%CV)	83.9 (5.43)	64.9 (14.1)	77.9 (12.6)

A = Peak area of aqueous mobile phase solutions without matrix and without extraction; B = Peak area of analyte spiked after extraction; C = Peak area of analyte spiked prior to extraction; %ME = Matrix effect expressed as the ratio of the mean peak area of the analyte spiked after extraction (B) to the mean peak area of an equivalent concentration of analyte in mobile phase (A) × 100; %RE = Extraction yield calculated as the ratio of the mean peak area of the analyte spiked prior to extraction (C) to the mean peak area of the analyte spiked after extraction (B) × 100; %PE = Process efficiency expressed as the ratio of the mean peak area of the analyte spiked prior to extraction (C) to the mean peak area of the same analyte in mobile phase (A) × 100; %CV coefficient of variation (standard deviation/mean × 100).

### 3.4 Dilution integrity

After 2× and 4× dilutions, the mean (%CV) back calculated concentrations for 9000 ng/mL DBS samples were within 92.4% (3.36) and 98.7% (9.13), 104.6% (3.94) and 114.6% (6.6) and 88.3% (3.36) and 94.1% (7.83) of their nominal values for 3TC, FTC and TFV, respectively.

### 3.5 Stability and re-injection reproducibility

Table 3 shows the short-term stability of 3TC, FTC and TFV in processed samples, and the long term stability in DBS and DBMS when stored at ambient temperature. Following re-injection of accepted validation batches of DBS and DBMS, stored in the autosampler for 24 h at 4 °C, all assay validation parameters were within acceptable limits, demonstrating re-injection reproducibility.

**Table 3:** Short and long term storage stability of DBS and DBMS (TFV, 3TF and FTC).

		Tenofovir		Lamivudine		Emtricitabine	
Storage condition	Level	DBS	DBMS	DBS	DBMS	DBS	DBMS
		Mean (%CV) [% acuaracy]	Mean (%CV) [% accuracy]	Mean (%CV) [% accuracy]	Mean (%CV) [% accuracy]	Mean (%CV) [% accuracy]	Mean (%CV) [% accuracy]
Autosampler stability of extracted samples (24 h at 4 °C)	LQC	42.8 (7.7) [107]	11.4 (7.8) [114]	38.1 (7.0) [95]	34.2 (5.4) [86]	39.6 (8.6) [99]	35.4 (5.5) [89]
	MQC	433 (4.3) [108]	40.3 (9.3) [101]	400.1 (1.9) [100]	377.8 (3.9) [94]	395 (4.1) [99]	371.5 (4.9) [93]
	HQC	3720 (6.4) [93]	554 (4.4) [111]	3820 (7.6) [96]	4177 (4.0) [104]	2964 (5.4) [74]	4314 (5.2) [108]
Long-term stability of dried blood spots (6 months at 24 °C)	LQC	41.7 (7.8) [104]		63.1 (6.9) [126]		44.7 (4.1) [111]	
	MQC	420 (8.8) [105]		433 (8.9) [108]		336.2 (5.6) [85]	
	HQC	4340 (6.2) [108]		4418 (4.5) [110]		3454.3 (12.1) [86]	
Long-term stability of dried milk spots (9 months at 24 °C)	LQC		10.9 (11.7) [109]		44.1 (6.1) [110]		44.8 (3.1) [112]
	MQC		43.9 (11.9) [110]		344 (3.9) [86]		411 (1.7) 103]
	HQC		466 (7.4) [104]		3655 (5.4) [91]		4207 (1.7) [105]

### 3.6 Effect of haematocrit

The diluted whole blood demonstrated HCT percentages of 55, 44, 31, 25, 16 and 0%. The effect of haematocrit in blood was negligible on the quantitation of 3TC, FTC and TFV concentrations in DBS until HCT fell to 8%, reflecting conditions of severe anaemia (Supplementary Figure A). In the patient population, the plasma:mDBS ratio for 3TC and TFV remained constant within haematocrit range 30–50% (Supplementary Figure B). All participants at both study sites had haematocrit within this range.

### 3.7 Correction for plasma

**Fig. 2:**
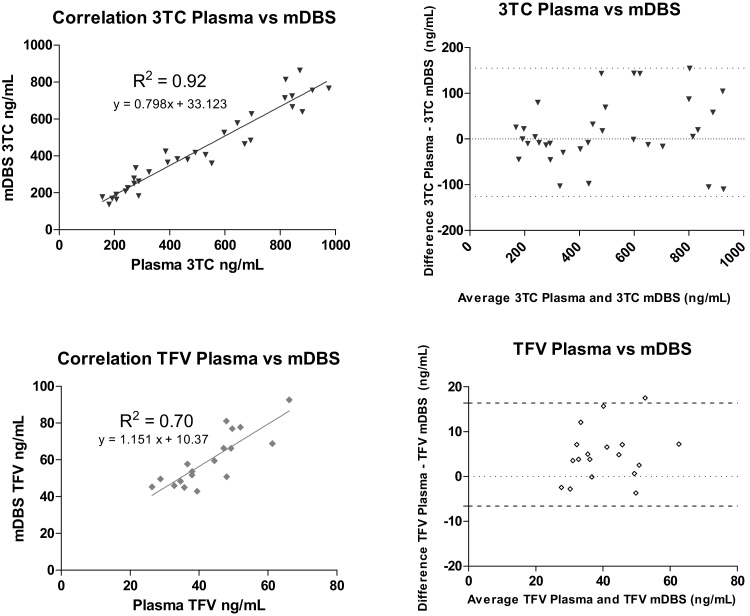
Correlation and Bland-Altman comparison between 3TC and TFV concentrations in mDBS and plasma. The dotted lines indicate the mean difference and the 95% limits of agreement (mean difference ± standard deviation)

Plasma and mDBS concentrations of 3TC and TFV showed a strong positive correlation (R2 = 0.92 and 0.70, respectively). Using a correction factor derived from the average plasma:mDBS ratio (0.88 for 3TC and 1.57 for TFV), Bland-Altman analysis indicated good agreement between the two methods (Fig. 2).

### 3.8 Clinical application

The six Ugandan mothers had a mean (range) age of 30 (24–41) years and a body weight of 54 (47–82) kg. Infants were aged 106 (83–146) days and weighed 5.88 (5.4–6.2) kg. In mDBS, 3TC reached a median (IQR) C_max_ of 993 (770–1598) at a T_max_ of 4 (2.5–5.5) h. The mDBS AUC_0-8_ was 4683 (4165–6057) ng h/mL. In BM, the C_max_ of 994 (958–1274) ng/mL was reached at a T_max_ of 3 (2–4) h, and the AUC_0-8_ was 6050 (5217–6417) ng h/mL. The DBMS:mDBS ratio of AUC_0-8_ was 1.31 (0.98–1.51). Two infants had detectable iDBS levels, of 13.2 and 15.6 ng/mL.

**Fig. 3:**
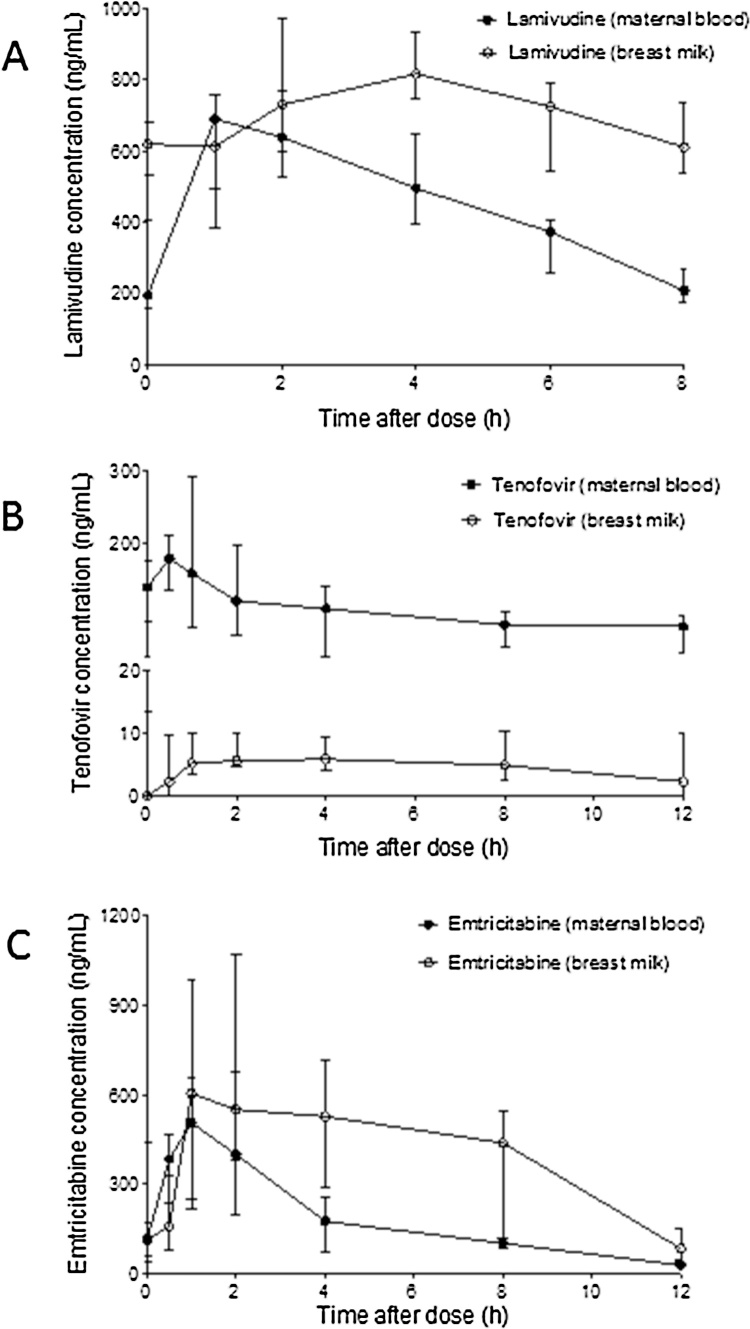
A Blood, BM and Infant 3TC concentrations at 0–8 h post 150 mg dose in 6 Ugandan mother-infant pairs; B Blood, BM and Infant FTC concentrations at 0–12 h post 300 mg dose in 6 Nigerian mother-infant pairs; C Blood, BM and Infant FTC concentrations at 0–12 h post 300 mg dose in 6 Nigerian mother-infant pairs. Data are presented as median (IQR)

The six Nigerian mothers had a mean age of 28 years (23–30) and weighed 58 (52–63) kg. Infants were of mean age 164 (101–219) days and weighed 7.5 (6–10) kg. In mDBS, TFV reached a C_max_ of 217 (181–278) ng/mL at a T_max_ of 2 (1.25–2) h, giving an AUC_0-12_ of 1559 (930–1915) ng h/mL. In the BM, a C_max_ of 5.9 (5.5–8.0) ng/mL was reached at a T_max_ of 3 (1–7) h, with an AUC_0-12_ of 56 (45–80) ng h/mL. The DBMS:mDBS ratio of AUC_0-8_ was 0.04 (0.025–0.058), and no infants had detectable iDBS levels. FTC in mDBS reached a C_max_ of 547 (467–719) at a T_max_ of 2 (1.25–3.5) h. The AUC_0-12_ was 3312 (2259–4312) ng h/mL. In BM, FTC reached a C_max_ of 872 (696–1063) at a T_max_ of 3 (2–4) h, and an AUC_0-12_ of 4853 (4124–6691) ng h/mL. The DBMS:mDBS ratio of AUC_0-12_ was 1.77 (1.62–2.24). One infant had detectable FTC at 17.5 ng/mL. These data are presented in Fig. 3.

## 4 Discussion

International guidelines now recommend that HIV positive women who are pregnant and breastfeeding receive triple antiretroviral therapy with EFV, TFV and 3TC or FTC [1]. To enable the further investigation of the pharmacokinetics of these drugs in this population, we have developed and validated a straightforward, accurate and precise method for the quantitation of 3TC, FTC and TFV in DBS and DMBS. Whilst recent work has described a DBS assay for TFV/FTC [20], [21], the method for 3TC quantitation in human DBS has not been reported, and DBMS assays for these NRTIs have not previously been developed. Furthermore, we report full pharmacokinetic profiles of plasma and BM FTC and TFV from HIV-positive breastfeeding mothers together with matched infant data.

Plasma processing requires relatively large volumes of blood, access to trained laboratory personnel and equipment for centrifugation and storage and specific shipping requirements which are cumbersome and expensive. DBS sampling therefore offers advantages of ease of collection, lower blood volumes, and simpler short-term storage and transport of specimens. This makes the technique ideal for field studies of vulnerable populations (such as pregnant women and breastfeeding infants). Whilst volumetric absorptive microsampling methodologies are emerging as an alternative method for accurate quantitation of drugs [22], these have not yet been widely used in low-resource settings. The method we report is immediately available and applicable for ongoing field studies.

In pilot cohorts of breastfeeding mother-infant pairs from Uganda and Nigeria, we have shown that both 3TC and FTC penetrate into the breast, with peak concentrations (described by both C_max_ and AUC) greater than those reached in maternal plasma. In two out of six Ugandan infants, 3TC was measurable, and in one out of six Nigerian infants, FTC could be quantified. On a programmatic level, this may have important implications. Whereas low levels of these drugs were initially thought to be of trivial clinical significance due to the level being lower than the reported inhibitory concentrations of the drug [23], more recent work indicated that the breastfed infants of HIV-positive mothers who require HIV despite maternal ART have high rates of multi-class drug resistance [4], [24] which is thought to relate to long term exposure to low levels of the drugs. Tenofovir has previously been measured in BM in cross-sectional studies [8], [9], [10] and we now report low but detectable concentrations in the BM of all six Nigerian women included in this pilot work. Although infant levels were not measurable, the clinical implications of low concentrations of tenofovir in the breast milk are not known.

Further evaluation of the pharmacokinetics of these drugs as WHO policy is implemented is therefore a priority to ensure the safest use of these drugs. Furthermore, clinical covariates which may impact on drug levels are often exclusion criteria from clinical and intensive pharmacokinetic studies; utilisation of sparse PK sampling to investigate the full spectrum of these patients will be informative, and we have now developed the necessary assay to facilitate such studies.

## Funding

CW was funded by an Academy of Medical Sciences Starter Grant for Clinical Lecturers subsequently by a Wellcome Trust Clinical Postdoctoral Training Fellowship WT104422MA.

AO received funding from the Tertiary Education Trust Fund, Nigeria and the University of Liverpool, UK.

## Transparency declarations

We acknowledge infrastructural support from the Liverpool Biomedical Research Centre funded by Liverpool Health Partners.

## Appendix A Supplementary data

Fig. S1 Impact of Haematocrit on Assay Stability. A: Reducing haematocrit concentrations generated by diluting donor blood with plasma prior to spiking with known concentration of drug. B: Ratio of plasma to DBS in samples from the same blood draw from study participants receiving ART.
