# Supplementary figures and images for: Development, validation and clinical application of a method for the simultaneous quantification of lamivudine, emtricitabine and tenofovir in dried blood and dried breast milk spots using LC–MS/MS
Source: J Chromatogr B Analyt Technol Biomed Life Sci. 2017 Aug 15;1060:300–7. doi: 10.1016/j.jchromb.2017.06.012 (PMC5588985; doi:10.1016/j.jchromb.2017.06.012)

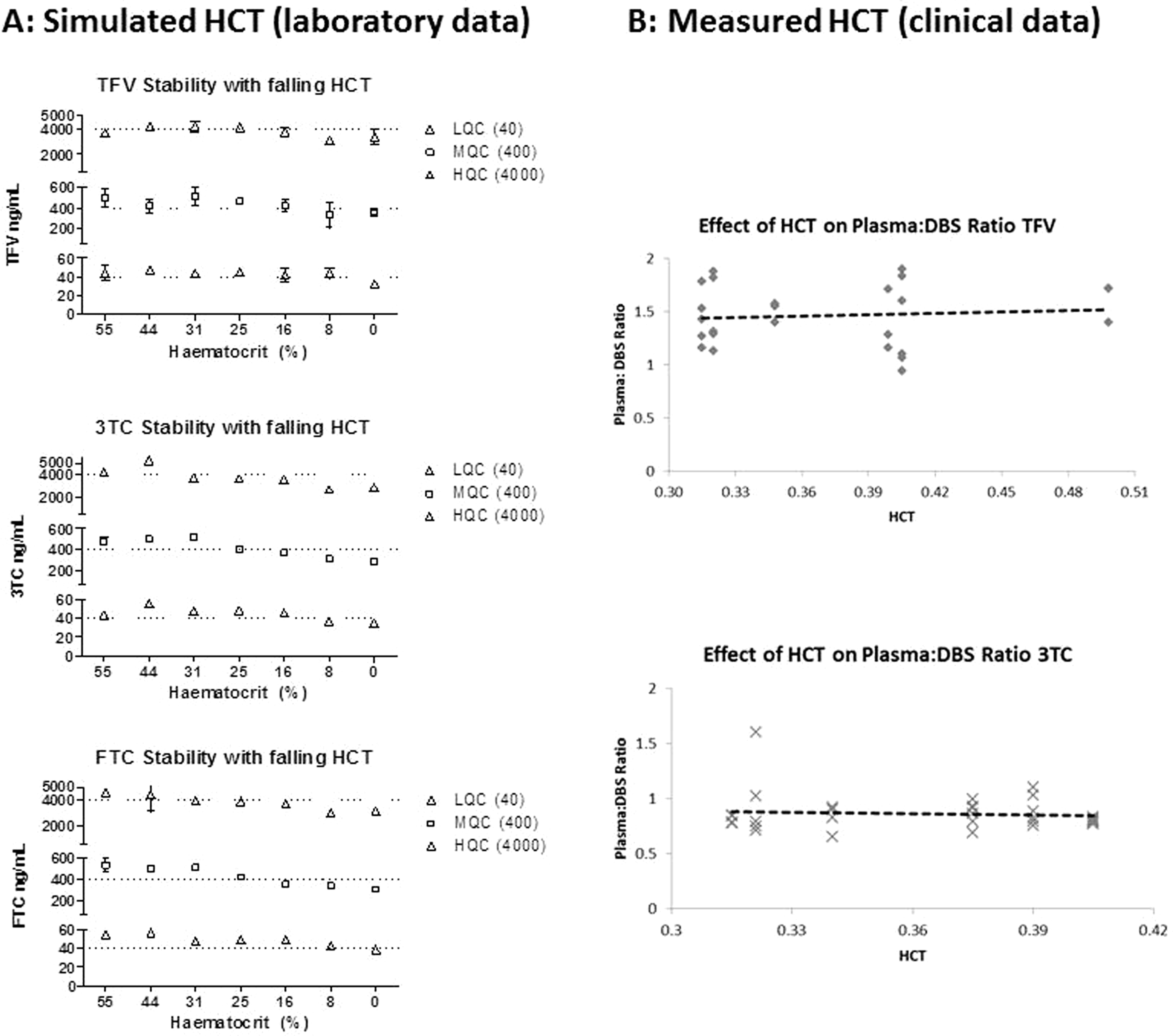

Supplement: Fig. S1 — Impact of Haematocrit on Assay Stability. A: Reducing haematocrit concentrations generated by diluting donor blood with plasma prior to spiking with known concentration of drug. B: Ratio of plasma to DBS in samples from the same blood draw from study participants receiving ART. [file mmc1.jpg]
